# O-serogroups, virulence genes, antimicrobial susceptibility, and MLST genotypes of Shiga toxin-producing *Escherichia coli* from swine and cattle in Central China

**DOI:** 10.1186/s12917-019-2177-1

**Published:** 2019-11-29

**Authors:** Zhong Peng, Wan Liang, Zizhe Hu, Xiaosong Li, Rui Guo, Lin Hua, Xibiao Tang, Chen Tan, Huanchun Chen, Xiangru Wang, Bin Wu

**Affiliations:** 1 State Key Laboratory of Agricultural Microbiology, College of Animal Science and Veterinary Medicine, Huazhong Agricultural University, Wuhan, 430070 China; 2 The Cooperative Innovation Center for Sustainable Pig Production, Huazhong Agricultural University, Wuhan, 430070 China; 3 Key Laboratory of Prevention and Control Agents for Animal Bacteriosis (Ministry of Agriculture), Animal Husbandry and Veterinary Institute, Hubei Academy of Agricultural Sciences, Wuhan, China

**Keywords:** Shiga toxin-producing *Escherichia coli*, O-serogroups, Virulence genes, Antimicrobial susceptibility, MLST genotypes

## Abstract

**Background:**

Shiga toxin-producing *Escherichia coli* (STEC) is a leading cause of worldwide food-borne and waterborne infections. Despite an increase in the number of STEC outbreaks, there is a lack of data on prevalence of STEC at the farm level, distribution of serogroups, and virulence factors.

**Results:**

In the present study, a total of 91 (6.16%) STEC strains were isolated from 1477 samples including pig intestines, pig feces, cattle feces, milk, and water from dairy farms. The isolation rates of STEC strains from pig intestines, pig feces, and cattle feces were 7.41% (32/432), 4.38% (21/480), and 9.57% (38/397), respectively. No STEC was isolated from the fresh milk and water samples. By O-serotyping methods, a total of 30 types of O-antigens were determined, and the main types were O100, O97, O91, O149, O26, O92, O102, O157, and O34. Detection of selected virulence genes (*stx*_1_, *stx*_2_, *eae*, *ehxA*, *saa*) revealed that over 94.51% (86/91) of the isolates carried more than two types of virulence associated genes, and approximately 71.43% (65/91) of the isolates carried both *stx*_1_ and *stx*_2_, simultaneously. Antimicrobial susceptibility tests showed that most of the STEC isolates were susceptible to ofloxacin and norfloxacin, but showed resistance to tetracycline, kanamycin, trimethoprim-sulfamethoxazole, streptomycin, amoxicillin, and ampicillin. MLST determined 13 categories of sequence types (STs), and ST297 (31.87%; 29/91) was the most dominant clone. This clone displayed a close relationship to virulent strains STEC ST678 (O104: H4). The prevalence of ST297 clones should receive more attentions.

**Conclusions:**

Our preliminary data revealed that a heterogeneous group of STEC is present, but the non-O157 serogroups and some ST clones such as ST297 should receive more attentions.

## Background

Shiga toxin-producing *Escherichia coli* (STEC) is a significant foodborne pathogen that is capable of causing watery or bloody diarrhea, hemorrhagic colitis, and hemolytic uremic syndrome [1–3]. O (somatic) polysaccharides and H (flagellar) surface antigens form the basis for the serological determination of STEC strains [4, 5]. There are currently more than 100 types of O antigens having been determined from STEC isolates, and several serogroups such as O157, O26, O104, O45, O103, O111, O121, and O145 are commonly associated with severe illness in humans worldwide [2, 4, 6–9]. In China, the first ever severe outbreak of *E. coli* O157:H7 occurred in Xuzhou, Jiangsu Province, in 1999, which caused the death of 177 people [10]. While limited data on STEC in humans in China are available, both STEC O157 and non-O157 STEC including some predominant serogroups associated with human disease, such as O26, O45, O103, O111, and O121, have been detected and isolated from domestic and wild animals as well as raw meats in different regions [11–14]. A recent study has revealed that the overall prevalence of STEC O157:H7 was 41.3% along the production and supply chain of pork around Hubei Province in Central China, and the prevalence found in slaughter houses, wet- and super-markets were 86.25% (69/80), 53.3% (32/60), and 28.3% (17/60), respectively [13]. These data suggest a big threat to the food safety and even human health in this region.

There are many virulence factors associated with the fitness and pathogenesis of STEC, but Shiga toxin (Stx, also called Vero toxin) is regarded as the most important one [1, 15]. STEC strains mainly produce two Stx types, Stx_1_ and Stx_2_, which are further classified into three subtypes for Stx_1_ (Stx_1a_, Stx_1c_, Stx_1d_) and seven subtypes for Stx_2_ (Stx_2a_, Stx_2b_, Stx_2c_, Stx_2d_, Stx_2e_, Stx_2f_, Stx_2g_) [16]. In addition to Stx, the STEC strains also possess many other virulence determinants, including the locus of enterocyte effacement (LEE), hemolysin, STEC autoagglutinating adhesion (Saa), lipopolysaccharide (LPS), outer membrane proteins (OMPs), fimbrial, and peroxidase [15, 17–22].

It is proposed that food-producing animals such as cattle, pigs, chickens are major reservoirs for STEC [23]; and many STEC outbreaks are associated with consumption of meat and other products of food-producing animals contaminated with STEC strains, and/or water contaminated with feces of food-producing animals [24, 25]. Despite an increase in the number of STEC outbreaks, there is a lack of data on prevalence of STEC at the farm level, distribution of serogroups, and virulence factors [2]. Since pork and milk are the common daily food for the Chinese people and Central China, including Hubei, Anhui, Hunan and Henan provinces, is one of main pig rearing and pork producing regions in China, in this study, we performed an isolation, identification and characterization of STEC strains from pigs, cattle, milk and water samples collected from pig and cattle farms in Central China.

## Results

### Isolation of STEC

**Fig. 1:**
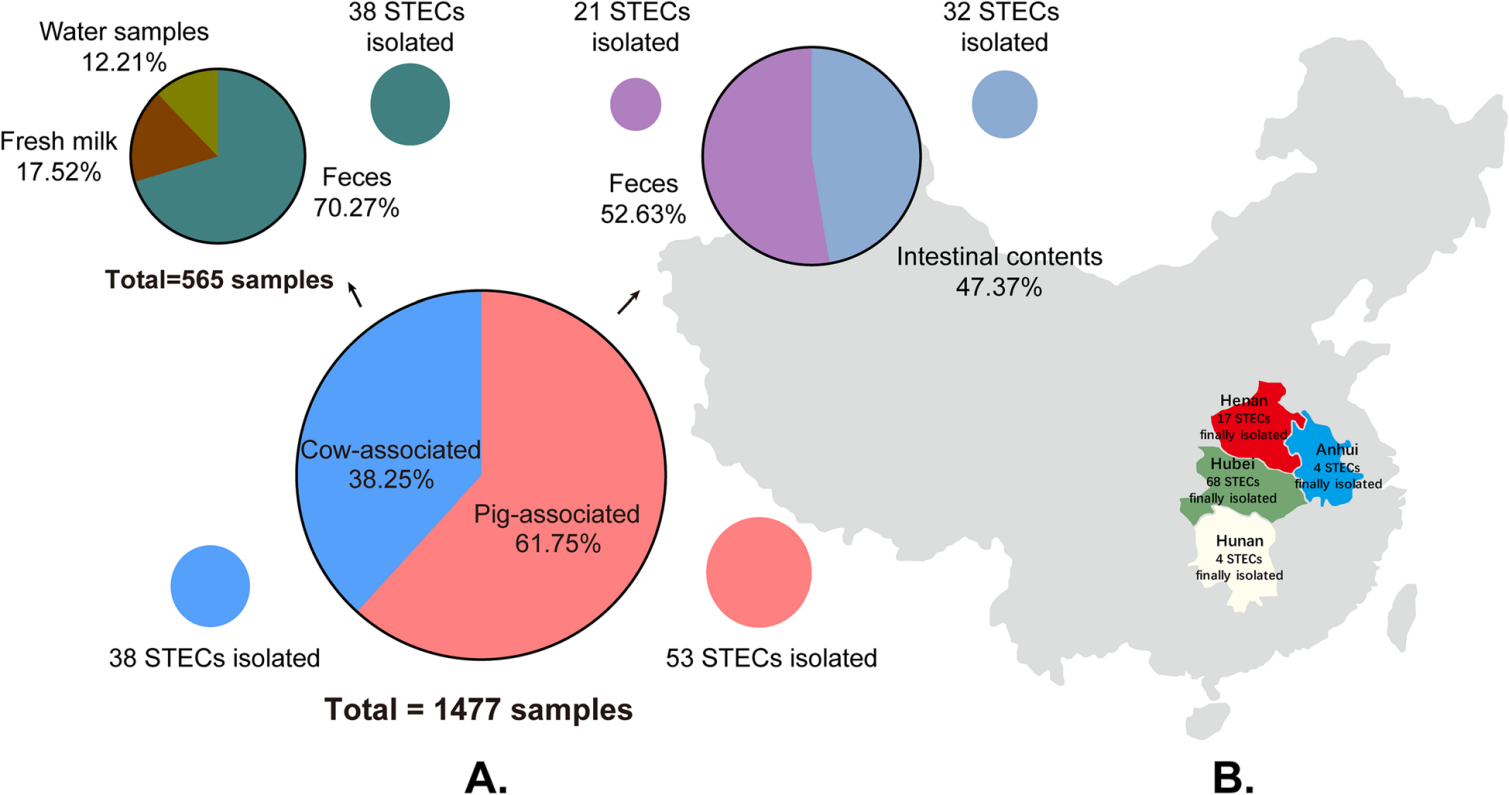
Isolation of STEC from samples collected from Hubei, Henan, Hunan, and Anhui in China. Panel **a** displays the number of each type of the samples collected for STEC isolation and the number of STEC strains isolated. Panel **b** shows the places of samples collected and the number of STEC isolated from different provinces in Central China. The authors sincerely acknowledge the Ministry of Natural Resources of the People’s Republic of China for providing the map of China free for public use

A total of 1477 samples, including 432 samples of intestinal contents from pigs with diarrhea, 480 fecal samples from pigs with diarrhea, 397 fecal samples from cows with diarrhea, 99 samples of fresh milk and 69 water samples from dairy farms, were collected from four provinces of Central China (Hubei, Anhui, Hunan, Henan) for PCR detection of Shiga toxin encoding genes (*stx*) and STEC isolation. Of the 1477 samples detected, 119 (8.06%) samples were positive for *stx*_1_ and/or *stx*_2_. STEC strains were isolated from 91 (76.47%) of the 119 *stx*-positive samples. The isolation rates of STEC strains from pig intestines, pig feces, and cattle feces were 7.41% (32/432), 4.38% (21/480), and 9.57% (38/397), respectively (Fig. 1a, Table 1). However, there were no STEC strains being isolated from the fresh milk and water samples collected (Fig. 1a, Table 1). Biochemical tests showed that all isolates were capable of fermenting glucose, maltose, lactose, and xylose, raffinose, lysine, and ornithine, but were unable to use gluconate, phenylalanine, and citrate.

**Table 1:** Serogroups, virulence factors and sequence types (STs) of the 91 STEC isolates

ST	No. of isolates	Serogroup	*Stx*_1_	*Stx*_2_	*eae*	*ehxA*	*saa*
ST10	5	O34	+	+	–	+	–
		O92	–	+	–	–	–
		O97	–	+	–	+	–
		O98	+	+	–	–	+
		O149	–	+	–	+	–
ST26	2	O69	+	+	–	+	+
		O100	–	+	–	+	+
ST29	11	O76	+	+	–	+	+
		O92	+	+	+	–	–
		O97	+	+	–	+	–
		O97	+	+	–	+	–
		O100	+	+	–	+	+
		O100	+	–	–	+	+
		O100	+	+	–	+	–
		O102	–	+	+	–	–
		O102	–	+	+	–	–
		O102	+	+	–	+	–
		Nontypable	+	+	–	+	–
ST101	7	O26	+	+	–	–	–
		O97	+	+	–	+	–
		O100	–	+	–	–	–
		O100	+	+	–	+	+
		O100	+	+	–	+	–
		O102	+	+	–	+	–
		O149	–	+	–	+	–
ST156	1	O64	+	+	–	+	–
ST297	29	O5	+	+	–	+	–
		O6	+	+	–	+	–
		O21	–	+	–	+	–
		O22	+	+	–	+	+
		O26	+	+	–	+	–
		O26	+	+	–	+	–
		O26	+	+	–	+	–
		O26	+	+	–	+	–
		O39	–	+	–	+	–
		O54	+	+	–	+	–
		O55	+	+	–	+	–
		O75	+	+	–	+	–
		O91	+	+	–	+	–
		O91	+	+	–	+	+
		O97	+	+	–	+	–
		O97	+	+	–	+	+
		O97	+	+	–	+	+
		O97	+	+	–	+	–
		O97	+	+	–	+	–
		O97	+	+	–	+	–
		O97	+	+	–	+	–
		O100	+	+	–	+	–
		O100	+	–	–	+	+
		O100	+	–	–	+	–
		O100	+	–	–	+	–
		O145	+	+	–	+	–
		O149	–	+	–	+	–
		O173	+	+	–	+	–
		O173	+	+	–	+	–
ST542	2	O157	+	+	+	+	–
		O157	+	+	+	+	–
ST602	13	O34	+	+	–	+	–
		O34	+	+	–	+	–
		O55	+	+	–	+	–
		O75	+	+	–	+	–
		O91	+	–	–	+	+
		O91	+	–	–	+	–
		O91	+	–	–	+	–
		O91	+	–	–	+	–
		O91	+	–	–	+	+
		O97	+	+	–	+	–
		O118	+	+	–	+	+
		O149	+	+	–	+	–
		Nontypable	+	+	–	+	–
ST793	1	O3	–	+	+	–	–
ST813	4	O34	+	+	–	–	–
		O92	+	+	–	+	–
		O97	+	+	–	+	–
		O100	+	+	–	+	–
ST1294	8	O91	+	–	–	+	+
		O100	+	+	–	+	+
		O110	+	+	–	+	+
		O149	+	+	–	+	–
		Nontypable	+	+	–	+	–
		Nontypable	+	+	–	+	–
		Autoagglutination	–	+	–	–	–
		Autoagglutination	–	+	–	–	–
ST1623	6	O42	+	+	–	+	–
		O54	+	+	–	+	–
		O78	–	+	–	+	+
		O92	+	+	–	+	+
		O92	+	+	–	+	+
		O149	–	+	–	–	–
ST1721	2	O9	+	+	–	+	+
		O167	+	+	–	+	+

“+”: Positive; “-”: Negative

### Serogroups and virulence genotypes

**Fig. 2:**
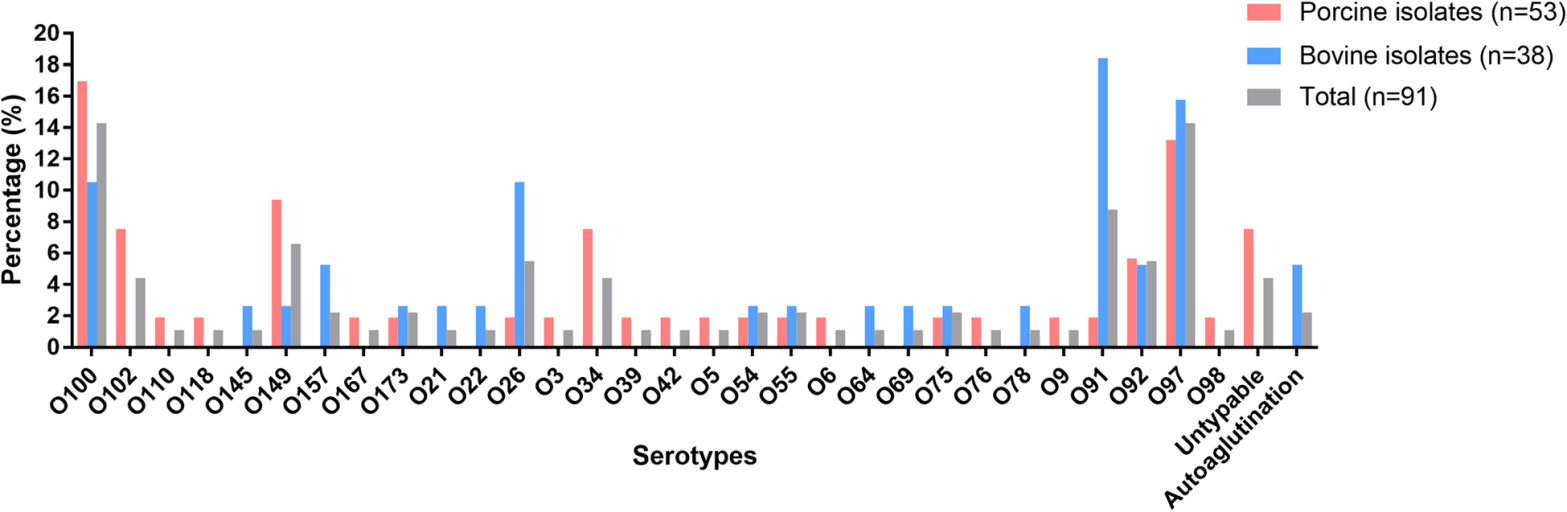
Distribution of the O-antigens among the STEC isolates. Columns in pink displays the percentage of STECs isolated from the feces and intestinal contents of pigs; Columns in sky blue displays the percentage of STECs isolated from the feces of cows; Columns in gray displays the percentage of the total STECs isolated herein (STECs from pigs plus STECs from cows)

By O-serotyping methods, a total of 30 categories of serogroups were determined for the 93 STEC isolates, and O100, O97, O91, O149, O26, O92, O102, O157, and O34 were the main serogroups (Fig. 2). There were 17 categories of serogroups identified among the bovine isolates (isolates from cow-associated samples), and 25 categories of serogroups among the porcine isolates (isolates from pig-associated samples) (Fig. 2, Table 1). Main serogroups among the porcine isolates were O100, O97, O149, O102, and O34. For bovine isolates, prevalent serogroups were O91, O97, O100, O157, and O26 (Fig. 2). In particularly, serogroup O157 was only detected in STEC strains originated from cows.

**Fig. 3:**
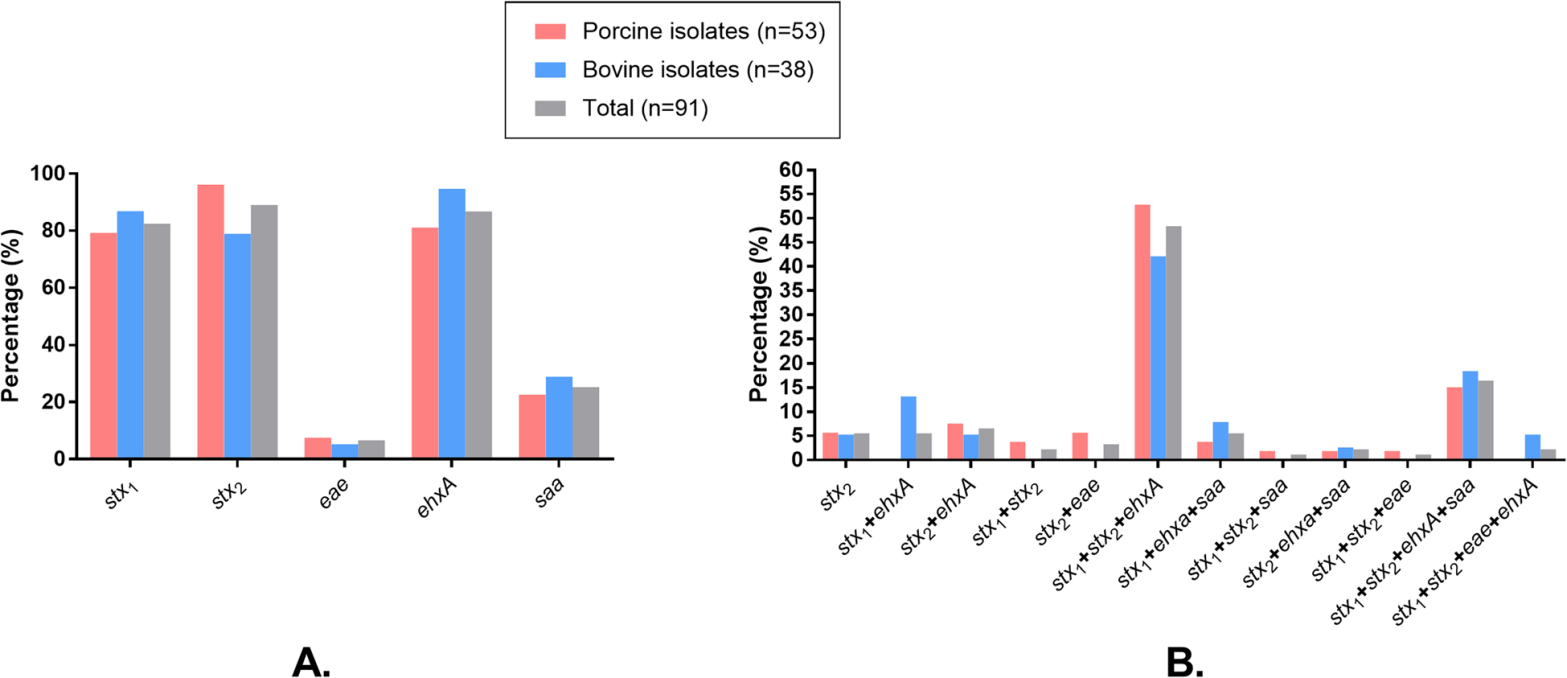
Distribution of main virulence genes (**a**) and their groups (**b**) among the STEC isolates. Panel **a** shows the distribution of main virulence genes while panel **b** shows the distribution of different groups of virulence genes. Columns in pink displays the percentage of STECs isolated from the feces and intestinal contents of pigs; Columns in sky blue displays the percentage of STECs isolated from the feces of cows; Columns in gray displays the percentage of the total STECs isolated herein (STECs from pigs plus STECs from cows)

The positive rate of the four virulence associated genes (*stx*_1_, *stx*_2_, *eae*, *ehxA*, *saa*) among the 91 STEC isolates ranged from 6.59% (*eae*, 6/91) to 89.01% (*stx2*, 81/91) (Fig. 3a). The detection rates of the two Stx encoding genes *stx*_1_ and *stx*_2_ were 82.42% (75/91) and 89.01% (81/91), respectively. Among the *stx*_1_-positive isolates, *stx*_1a_ was the most predominant subtype (78.67%, 59/75), followed by *stx*_1c_ (17.33%, 13/75) and *stx*_1d_ (4.00%, 3/75). For the *stx*_2_-positive isolates, *stx*_2e_ was the most predominant subtype (56.79%, 46/81), followed by *stx*_2b_ (17.28%, 14/81), *stx*_2d_ (9.88%, 8/81), *stx*_2a_ (7.14%, 6/81), *stx*_2c_ (6.17%, 5/81) and *stx*_2g_ (2.47%, 2/81). *Stx*_1a_ (100%, 33/33) and *stx*_2b_ (46.67%, 14/30) were the most predominant *stx*_1_ and *stx*_2_ subtypes for the bovine isolates while *stx*_1a_ (61.90%, 26/42) and *stx*_2e_ (91.20%, 46/51) were the most predominant *stx*_1_ and *stx*_2_ subtypes for the porcine isolates. Over 94.51% (86/91) of the isolates carried more than two types of virulence associated genes, and approximately 71.43% (65/91) of the isolates carried both *stx*_1_ and *stx*_2_, simultaneously (Fig. 3b). The percentages of the isolates carrying four types, three types, two types, and one type of the virulence genes detected were 18.68% (17/91), 58.24% (53/91), 17.58% (16/91), and 5.49% (5/91), respectively. Approximately 10.99% (10/91) of the isolates only carried *stx*_1_, and 17.58% (16/91) of the isolates only carried *stx*_2_.

In combination the serogroups with the virulence genes, isolates with different serogroups except O157 carried at least one Stx encoding gene (Table 1). In addition, all O100, O149, O26, O34, O91, and O97 isolates (the number of isolates with these serogroups is more than three) were negative to *eae*, while all O102, O149, O157, O26, and O34 isolates were PCR-negative for the presence of *saa* (Table 1).

### Cytotoxicity

Cytotoxicity tests showed that all isolates positive to *stx*_1_ and/or *stx*_2_ were capable of making Vero cells rounding and exfoliation. However, no cytopathic effect was observed in the cells inoculated with the isolates negative to both *stx*_1_ and *stx*_2_.

### Antimicrobial susceptibility

**Fig. 4:**
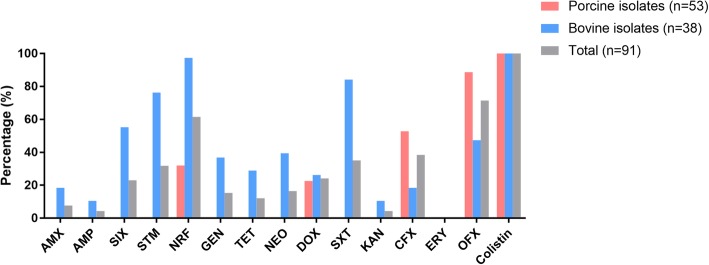
Antimicrobial susceptibility of the 91 STEC isolates. AMX: amoxicillin; AMP: ampicillin; SIX: sulfafurazole; STM: streptomycin; NRF: norfloxacin; GEN: gentamicin; TET: tetracycline; NEO: neomycin; DOX: doxycycline; SXT: trimethoprim-sulfamethoxazole; KAN: kanamycin; CFX: cefotaxime; ERY: erythromycin; OFX: ofloxacin. Columns in pink displays the percentage of STECs isolated from the feces and intestinal contents of pigs; Columns in sky blue displays the percentage of STECs isolated from the feces of cows; Columns in gray displays the percentage of the total STECs isolated herein (STECs from pigs plus STECs from cows)

Antimicrobial susceptibility testing results showed that more than 50% of the STEC isolates were sensitive to ofloxacin (71.43%; 65/91), and norfloxacin (61.54%; 56/91). However, less than 10% of the isolates were sensitive to amoxicillin (7.69%; 7/91), ampicillin (4.40% 4/91), and kanamycin (4.40% 4/91). In particularly, all isolates were resistant to erythromycin (100%, 91/91) (Fig. 4). Most of the isolates from cattle feces were sensitive to norfloxacin (97.37%; 37/38), trimethoprim-sulfamethoxazole (84.21%, 32/38), and streptomycin (76.32%; 29/38). All isolates tested herein were sensitive to colistin; the MIC values were determined as ≤1 μg/ml. Approximately half of the bovine isolates were sensitive to sulfafurazole (55.26%; 21/38) and ofloxacin (47.37%; 18/38). For isolates from pigs, 88.68% (47/53) of the isolates were sensitive to ofloxacin. However, there were no isolates from pig intestines and/or feces sensitive to trimethoprim-sulfamethoxazole, streptomycin, sulfafurazole, neomycin, gentamicin, tetracycline, amoxicillin, ampicillin, kanamycin, and cefotaxime (Fig. 4).

### MLST genotypes

**Fig. 5:**
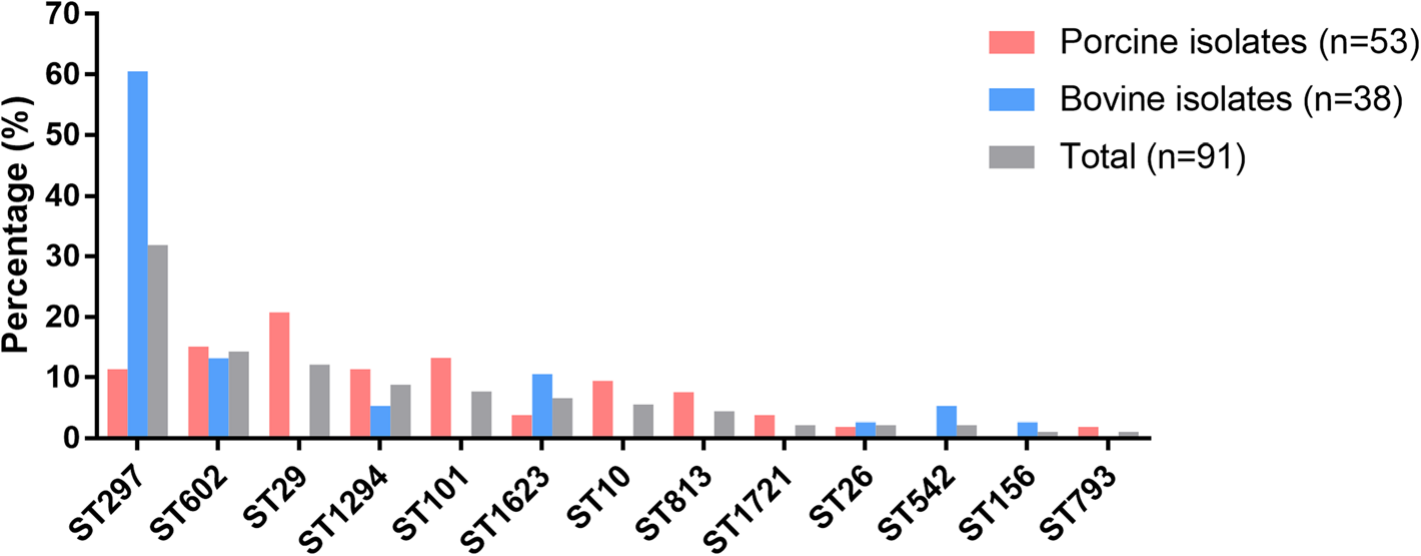
Distribution of the sequence types (STs) among the STEC isolates. Columns in pink displays the percentage of STECs isolated from the feces and intestinal contents of pigs; Columns in sky blue displays the percentage of STECs isolated from the feces of cows; Columns in gray displays the percentage of the total STECs isolated herein (STECs from pigs plus STECs from cows)

A total of 13 categories of sequence types (STs) were determined among the 91 STEC isolates using the MLST method (Fig. 5). Among these STs, ST297 (31.87%; 29/91) was the most frequent, followed by ST602 (14.29%; 13/91). The other determined STs included ST29 (12.09%; 11/91), ST1294 (8.79%; 8/91), ST101 (7.69%; 7/91), ST1623 (6.59%; 6/91), ST10 (5.49%; 5/91), ST813 (4.39%; 4/91), ST542 (2.20%; 2/91), ST1721 (2.20%; 2/91), ST26 (2.20%; 2/91), ST156 (1.10%; 1/91), and ST793 (1.10%; 1/91). For the 53 porcine isolates, a total of 11 types of STs were determined, and ST29 (20.75%; 11/53), ST602 (15.09%; 8/53), ST101 (13.21%; 7/53), ST297 (11.32%; 6/53), and ST1294 (11.32%; 6/53) were the common STs (Fig. 5). For the 38 bovine isolates, seven types of STs were identified, and ST297 (60.53%; 23/38), ST602 (13.16%; 5/38), ST542 (5.26%; 2/38), and ST1623 (10.53%; 4/38) were commonly present (Fig. 5).

**Fig. 6:**
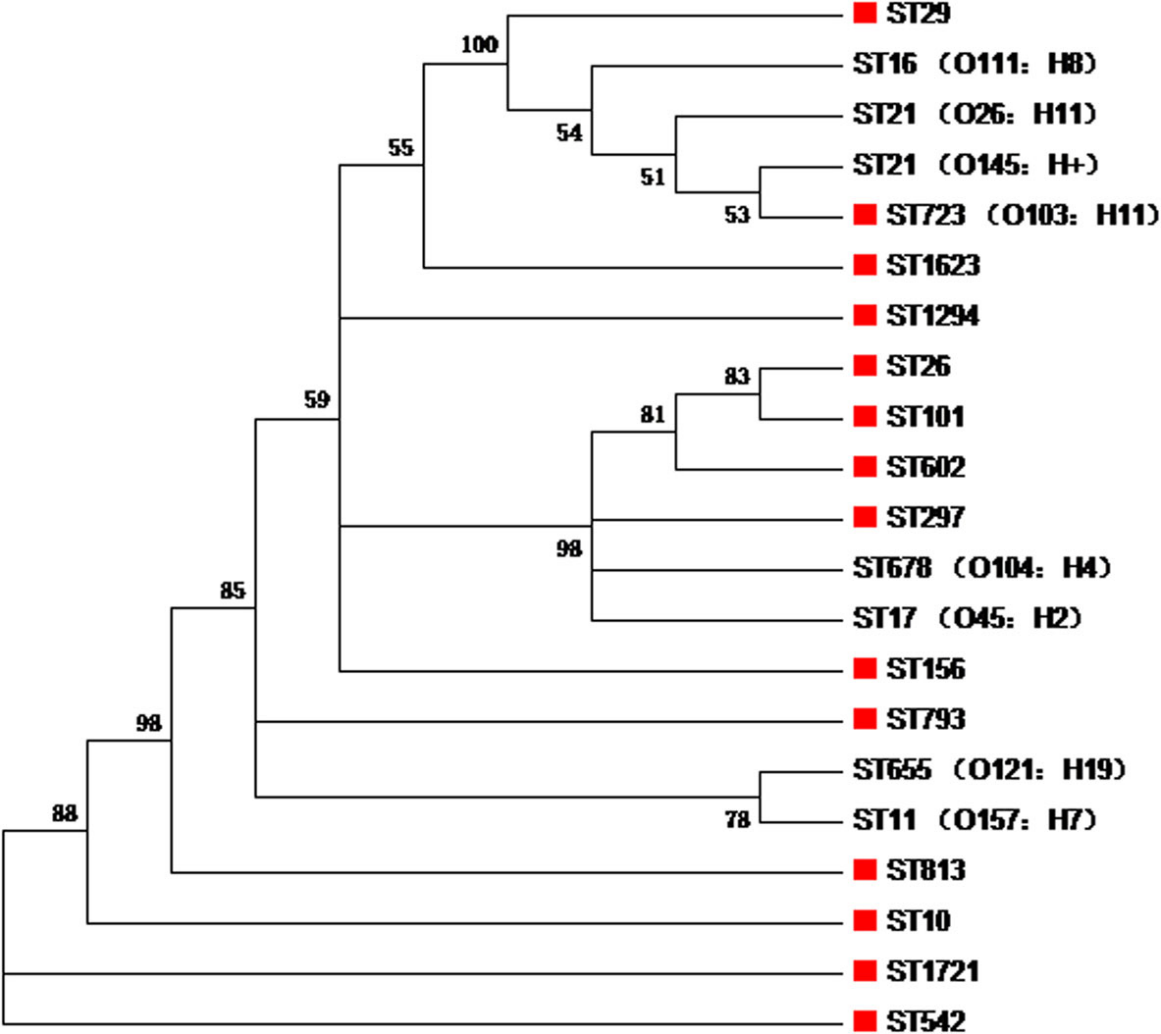
Phylogenetic analysis on different STEC sequence types clones. The tree was constructed based on the MLST data by MEGAX [26], using neighbor-joining algorithm with 1000 bootstrapping

Phylogenetic analysis showed that the MLST genotypes ST297, ST602, ST101, and ST26 displayed a relationship, and they also showed a close relatedness to the epidemic MLST genotypes ST678 (O104: H4) and ST17 (O45: H2) (Fig. 6). In addition, genotype ST29 was closely related to ST16 (O111: H8), ST21 (O26: H11; O145: H+), and ST723 (O103: H11) (Fig. 6).

## Discussion

STEC is a leading cause of foodborne and waterborne infections worldwide, food-producing animals such as cattle, and pigs are major reservoirs for STEC [23]. Among different kinds of food producing animals, cattle and other ruminants are considered to be the major reservoirs for STEC [23, 27, 28]. STEC strains are more frequently isolated from cattle and other ruminants than from other animals such as pigs, cats, and dogs [23, 29]. In agreement with these suggestions, the rate of STEC isolation from cattle (9.57%) was higher than that from pigs (5.81%). However, the isolate rate of STEC from cattle feces (9.57%) in present study is different from the reports from the other countries [2, 29]. These differences might be explained by differences in feed, seasonal peak, age, or detecting methods [2].

A total of 30 types of O-antigen were determined for the 91 STEC isolates by O-serotyping methods, with the exception of 4 isolates which were not typable (Fig. 2). This might be because there are only 50 types of O antisera are available, and these four strains do not react with the available antisera. Among these 30 categories of O-serogroups, the most frequently occurring serogroups were O100, O97, O91, O149, O26, O92, O102, O157, and O34 (Fig. 2). These serogroups have been isolated from pigs, cattle, sheep, and water in both China and the other countries [30–37]. It has been known that O157 is the most common serogroup that causes human illness in most parts of the world [4]. It has been also reported that cattle are the most common reservoir of *E. coli* O157 [38]. Corresponding to this suggestion, the O157 serogroup was only determined within the isolates from cattle in the present study (Fig. 2). Although there was no STEC O157 being isolated from pigs in Central China in the present study, a recent study has revealed that the overall prevalence of *E. coli* O157:H7 in pig farms around Hubei, a province located in Central China, is approximately 12.8% (16/125) [13]. These findings suggest that the prevalence of *E. coli* O157 in this region is still a problem. In addition to O157, O26 also displayed a high proportion of identification (Fig. 2). It is worthy of note that this type of O-antigen has been declared by the U.S. Department of Agriculture (USDA) as one of the “Big 6” (O26, O45, O103, O111, O121, and O145) non-O157 serogroups that are most commonly associated with severe illness in humans [4]. STEC O26 has been detected and isolated from diarrheal patient in China [11]. It should be noted that another member of the “Big 6”, the O145, was also identified in the present study (Fig. 2). In addition, STEC O149 has been also detected and isolated from diarrheal patient in China [11]. The determination of these non-O157 serogroups represents a great risk on public health and should also receive more attentions.

Virulence genotyping based on the detection of six virulence genes (*stx*_1_, *stx*_2_, *ehxA*, *eae*, and *saa*) showed that the detection rates of *stx*_1_ (82.42%), *stx*_2_ (89.01%), and *ehxA* (86.81%) were higher than those of the other virulence genes (Fig. 3a); most of the STEC isolates possess *stx*_1_, *stx*_2_, and *ehxA* simultaneously (Fig. 3b). It is known that both *stx*_1_ and *stx*_2_ are responsible for encoding the Shiga toxin, which is the most important and common virulence factors of STEC [15]. In particular, the detection rate of *stx*_2_ (89.01%) was higher than *stx*_1_ (82.42%), and a small proportion of isolates (5.49%, 5/91) only carried *stx*_2_ (Fig. 3a and b). It has been reported that *stx*_2_ is more often associated with severe disease [39]. Therefore, those strains might be more harmful. Both Stx_1_ and Stx_2_ have several subtypes, and some subtypes are more frequently associated with human disease [4]. It has been widely documented that STEC isolates from pigs normally harbor Stx_2e_ subtype [40–42], and in agreement with these studies [40–42], approximately 91.20% of the porcine isolates positive to *stx*_2_ determined in the present study harbored this subtype (Stx_2e_). STEC producing Stx2e is known to be closely associated with edema disease in pigs [43], the high proportion of *stx*_2e_ detection in STEC isolates from pig intestines and/or feces in this study suggest a big threat to the pig health. Although Stx_2e_-producing STEC strains are still not proposed as pathogens for humans [43], active actions are still required to control and decrease the prevalence of such strains in pigs in a One Health perspective. In the present study, we also identified several other Stx-subtypes such as Stx_1a_, Stx_1c_, Stx_1d_, Stx_2b_, Stx_2d_, Stx_2a_, Stx_2c_, and Stx_2g_. Among these subtypes, *stx*_2a_ and *stx*_2c_ are proposed to be associated with high virulence and the ability to cause hemolytic-uremic syndrome (HUS), while *stx*_2d_, *stx*_2e_, *stx*_1a_, and *stx*_1c_ occurred in milder or asymptomatic infections [43, 44]. The detection of those subtypes in STEC strains from food producing animals such as pigs and cows we detected in this study represents a high risk on public health. It is worthy of note that *stx*_1d_, *stx*_2b_, *stx*_2g_ have been also detected in STEC strains from patients in Demark, however, HUS does not develop in these patients [45–47].

In addition to *stx*_1_ and *stx*_2_, the prevalence of *ehxA* (86.81%) was also very high, showing a good agreement with previously studies [48–51]. It is worthy of note that *ehxA* is generally used as a diagnostic indicator because the presence of *ehxA* is frequently correlated with the Shiga toxin [49, 51]. In agreement with this conclusion, *ehxA* displayed a high detection rate from the *stx*-positive STEC strains in the present study (Fig. 3b). In contrast to these genes which have high rates of detection, the detection rates of *eae*, and *saa* were relatively low. These results are similar to previously studies [50, 51], suggesting that these virulence genes are not common. However, their presence in particularly the detection of *eae* should be given a concern. It has been reported that the combination of *eae* and *stx*_2_ has an especial association with the development of HUS and bloody diarrhea [46, 47, 52]. In the present study, all *eae*-positive STEC strains isolated in Central China were detected to be positive for *stx*_2_ (Table 1). The determination of such strains represents a high risk on public health in this region.

The antimicrobial resistance (AMR) of STEC is also a serious problem that the world is now facing. It has been reported that STEC isolates from both humans and food-producing animals displayed resistance most often to tetracycline, kanamycin, trimethoprim-sulfamethoxazole, streptomycin, amoxicillin, and ampicillin [36, 53–56]. In agreement with these studies, a low proportion of STEC isolates from the present study was susceptible to those types of antimicrobials (Fig. 4). These findings suggest a serious profile of AMR in STEC in food-producing animals. While there is a number of articles reporting the colistin resistance prevalence in *E. coli* [57–59], it is worthy of note that all STEC isolates were sensitive to colistin in the present study.

MLST is also a strategy commonly used for STEC surveillance [36, 60]. In this study, 13 types of STs were determined for the 91 STEC isolates. In particularly, many isolates belonging to different STs possessed the same serogroups (Table 1). These findings are consistent with the findings of other publications [61, 62], suggesting that STEC isolates with the same serogroups might have genotypical diversity. Among the determined STs, ST297 possesses the highest rate of isolation (31.87%) compared to the remaining identified STs (Fig. 5). Interestingly, ST297 is rarely reported in STEC. A previous study determined five ST297 from 75 STEC food strains, with a detection rate of 6.67% [63]. In another study, the detection rate of ST297 among STEC isolates from cattle in Korea was only 4.69% (3/64) [64]. Our results are quite different from these studies, suggesting that the prevalence of the ST in different regions of the world might be different. The ST297 isolates harbored many types of O-antigens, including O26 and O145, the important members of the “Big 6” declared by the USDA [4]. In particular, all O26 isolates recovered in the present study are ST297 clone (Table 1). It has been reported that the STs of STEC O26 associated with a broad spectrum of diseases in Europe are ST29 and/or ST21 [65–70]. In addition, the ST297 clones isolated in this study displayed a close relationship to STEC ST678 (O104: H4) (Fig. 6). It should be noted that the STEC ST678 (O104: H4) isolates have caused the outbreak of human gastroenteritis and human hemolytic-uremic syndrome in Europe [8, 71]. In the next step, we intend to do follow up study to determine the genetic and phenotypical characteristics of these ST297 clones. In addition, the sequence types of the two STEC O157 were determined as ST542 (Table 1). Although the sequence type of STEC O157 is normally determined as ST11 [61, 72], O157 isolates determined as non-ST11 have been also documented elsewhere. For instance, four O157 isolates from the US and/or UK are determined as ST1804 [62]. These findings suggest there might be other STs for STEC O157. In the next step, we will do follow up study to determine the genetic and phenotypical characteristics of these two isolates.

## Conclusions

In conclusion, the present study performed an isolation and a characteristic analysis of STEC from pigs and cattle. Our preliminary data revealed that a heterogeneous group of STEC is present, but the non-O157 serogroups and some ST clones such as ST297 should receive more attentions. In the next step, we intend to do a follow up study to correlate the pathogenicity of these STEC with the *stx*-subtypes as well as the ST clones.

## Methods

### Sample collection and bacterial isolation

A total of 1477 samples were tested in this study. These samples included intestinal contents from pigs with diarrhea (432 samples), fecal samples from pigs (480 samples) and cows (397 samples) with diarrhea, fresh milk (99 samples), and water samples from dairy farms (69 samples) (Fig. 1a). The 912 pig-associated samples (feces and intestinal contents) were collected from 323 pig farms in Central China (Hubei, Anhui, Hunan, Henan) between 2016 and 2017, while the 565 cow-associated samples (feces, milk, and water samples from dairy farms) were from three dairy farms in different regions Hubei Province in 2017 (Fig. 1a and b). Bacterial isolation was performed following a previously described protocol with some modifications [2]. In brief, each of the samples were mixed in sterilized 0.9% normal saline by vortexing. After a centrifugation at 500×*g* for 1 min, 500 μL of the supernatant of the mixture was inoculated into 5 mL modified *E. coli* broth (Nissui, Tokyo, Japan) and incubated at 37 °C for 18~24 h.

After that, genomic DNA was extracted from the cultures by boiling 100 μL aliquot of each incubated broth directly, as described previously [2]. The extracted DNA was evaluated by electrophoresis on a 1% agarose gel and/or using a Nanodrop2000 (Thermo Scientific, Waltham, USA). Presence of the Stx encoding gene *stx*_1_ and/or *stx*_2_ was determined by PCR assays using the genomic DNA extracted herein as the template and the primers listed in Table 2. PCR reaction was performed in a 25 μL mixture containing 2 μL of the template DNA, 2.5 μL of 10× PCR Buffer (TAKARA, Japan), 2 μL of dNTP (TAKARA, Japan), 0.5 μL of rTaq (TAKARA, Japan), each of the forward and reverse primers 0.5 μL, and 17.0 μL of nucleotide-free water (TAKARA, Japan). Thermocycler conditions used for PCR were 95 °C for 5 min, followed by 30 cycles of denaturation at 94 °C for 30 s, annealing at different temperatures listed in Table 2 for 40 s, and extension at 72 °C for 1 min, with a final extension at 72 °C for 10 min before storage at 4 °C. DNA from STEC O157:H7 strain EDL933 and nucleotide-free water were included as positive and blank controls, respectively. The PCR product was visualized using 1% agarose gel electrophoresis under ultraviolet light.

**Table 2:** Primers used in this study

Primers	Sequences (5′-3′)	Annealing Temp. (°C)	Product size (bp)	Function	References
Bacterial identification and virulence genotyping					
stx_1_-F	ACACTGGATGATCTCAGTGG	60	614	Amplifying *stx1*	Botteldoorn et al., 2003^b^
stx_1_-R	CTGAATCCCCCTCCATTATG				
stx_2_-F	GGCACTGTCTGAAACTGCTCC	64	255	Amplifying *stx2*	Leung et al., 2001^d^
stx_2_-R	TCGCCAGTTATCTGACATTCTG				
16S-F	ATGGCTCAGATTGAACGC	50	1505	Amplifying *16 SrRNA*	REN et al., 2012^g^
16S-R	CAGGTTCCCCTACGGTTA				
eae-F	GTGGCGAATACTGGCGAGACT	64	890	Amplifying *eae*	Nielsen et al., 2003^e^
eae-R	CCCCATTCTTTTTCACCGTCG				
ehxA-F	GCATCATCAAGCGTACGTTCC	60	534	Amplifying *ehxA*	Bandyopadhyay et al., 2011^a^
ehxA-R	AATGAGCCAAGCTGGTTAAGCT				
saa-F	CCTCACATCTTCTGCAAATACC	60	1688	Amplifying *saa*	Paton et al., 2001^f^
saa-R	GTTGTCGTTCATATTTTACCATCCAATGGACATG				
MLST genotyping					
Adk-F1	TCATCATCTGCACTTTCCGC	54	583	Amplifying *adk*	Ding et al., 2012^c^
Adk-R1	CCAGATCAGCGCGAACTTCA				
FumC-F1	TCACAGGTCGCCAGCGCTTC	54	806	Amplifying *fumC*	
FumC-R1	GTACGCAGCGAAAAAGATTC				
GyrB-F1	TCGGCGACACGGATGACGGC	60	911	Amplifying *gyrB*	
GyrB-R1	ATCAGGCCTTCACGCGCATC				
Icd-F1	ATGGAAAGTAAAGTAGTTGTT CCGGCACA	54	878	Amplifying *icd*	
Icd-R1	GGACGCAGCAGGATCTGTT				
Mdh-F1	ATGAAAGTCGCAGTCCTCGGC GCTGCTGGCGG	60	932	Amplifying *mdh*	
Mdh-R1	TTAACGAACTCCTGCCCCAGAGCGATATCTTTCTT				
PurA-F1	TCGGTAACGGTGTTGTGCTG	54	816	Amplifying *purA*	
PurA-R1	CATACGGTAAGCCACGCA GA				
RecA-F1	CGCATTCGCTTTACCCTGACC	58	780	Amplifying *recA*	
RecA-R1	TCGTCGAAATCTACGGACCGGA				
Adk-F2	TCATCATCTGCACTTTCCGC	**–**	**–**	*adk* Sequencing	
Adk-R2	CCAGATCAGCGCGAACTTCA				
FumC-F2	TCACAGGTCGCCAGCGCTTC	**–**	**–**	*fumC* Sequencing	
FumC-R2	TCCCGGCAGATAAGCTGTGG				
GyrB-F2	TCGGCGACACGGATGACGGC	**–**	**–**	*gyrB* Sequencing	
GyrB-R2	GTCCATGTAGGCGTTCAGGG				
Icd-F2	ATGGAAAGTAAAGTAGTTGTTCCGGCACA	**–**	**–**	*icd* Sequencing	
Icd-R2	GGACGCAGCAGGATCTGTT				
Mdh-F2	AGCGCGTTCTGTTCAAATGC	**–**	**–**	*mdh* Sequencing	
Mdh-R2	CAGGTTCAGAACTCTCTCTGT				
PurA-F2	CGCGCTGATGAAAGAGATGA	**–**	**–**	*purA* Sequencing	
PurA-R2	CATACGGTAAGCCACGCAGA				
RecA-F2	ACCTTTGTAGCTGTACCACG	**–**	**–**	*recA* Sequencing	
RecA-R2	TCGTCGAAATCTACGGACCGGA				

^a^Bandyopadhyay S, Mahanti A, Samanta I, Dutta TK, Ghosh MK, Bera AK, Bandyopadhyay S, Bhattacharya D. Virulence repertoire of Shiga toxin-producing Escherichia coli (STEC) and enterotoxigenic Escherichia coli (ETEC) from diarrhoeic lambs of Arunachal Pradesh, India. Trop Anim Health Prod. 2011;43(3):705-10

^b^Botteldoorn N, Heyndrickx M, Rijpens N, Herman L. Detection and characterization of verotoxigenic Escherichia coli by a VTEC/EHEC multiplex PCR in porcine faeces and pig carcass swabs. Res Microbiol. 2003;154(2):97-104

^c^Ding Y, Tang X, Lu P, Wu B, Xu Z, Liu W, Zhang R, Bei W, Chen H, Tan C. Clonal analysis and virulent traits of pathogenic extraintestinal Escherichia coli isolates from swine in China. BMC Vet Res. 2012;8:140

^d^Leung PH, Yam WC, Ng WW, Peiris JS. The prevalence and characterization of verotoxin-producing Escherichia coli isolated from cattle and pigs in an abattoir in Hong Kong. Epidemiol Infect. 2001;126(2):173-9

^e^Nielsen EM, Andersen MT. Detection and characterization of verocytotoxin-producing Escherichia coli by automated 5' nuclease PCR assay. J Clin Microbiol. 2003;41(7):2884-93

^f^Paton AW, Srimanote P, Woodrow MC, Paton JC. Characterization of Saa, a novel autoagglutinating adhesin produced by locus of enterocyte effacement-negative Shiga-toxigenic Escherichia coli strains that are virulent for humans. Infect Immun. 2001;69(11):6999-7009

^g^REN L, YU X, SONG D, ZHEN K, QIN Y, WANG Y. Isolation, identification and phylogenetic analysis of E. coli from Yaks. China Animal Husbandry & Veterinary Medicine. 2012;39(1): 168-171

In the next step, bacterial cultures positive to at least one of *stx*_1_ and *stx*_2_ were streak-plated onto sorbitol MacConkey agar (Hangzhou Microbial Reagent CO., LTD, Hangzhou, China), and incubated at 37 °C for 18~24 h. After this stage, the isolates were purified and cultured following the standard methods used for bacterial identification [73]. Presumptive isolates of *E. coli* were finally confirmed via Galanz staining, biochemical testing, and 16S rRNA amplification and sequencing.

### Serotyping and virulence genotyping

O-polysaccharide antigens serogroups of STEC isolates were determined by Slide agglutination test based on the reaction of the bacterial strains against the 50 kinds of O antisera purchased from China Institute of Veterinary Drug Control (Beijing, China). STEC O157:H7 strain EDL933 was used as positive control.

Virulence genotyping was performed by PCR assays amplifying another three virulence associated genes *eae*, *ehxA*, and *saa* of with primers listed in Table 2. The PCR volume and procedure were the same as that used for determining the Stx encoding genes. Positive and blank control samples were included in each set of reactions. The PCR product was visualized using 1% agarose gel electrophoresis under ultraviolet light. The *stx* subtypes (*stx*_1a_, *stx*_1c_, *stx*_1d_, *stx*_2a_, *stx*_2b_, *stx*_2c_, *stx*_2d_, *stx*_2e_, *stx*_2f_, *stx*_2g_) were also determined by PCR assays with primers and reaction procedures described previously [16].

### Cytotoxicity

Vero cells (purchased from ATCC) were used to test the cytotoxicity of the STEC strains isolated herein. In brief, isolates were inoculated in Luria-Bertani (LB) broth (Sigma-Aldrich, MO) and shaken at 37 °C for 18~24 h. Bacterial culture were then centrifuged at 20000×*g* for 40 min, followed by a filtration through a 0.22 μm membrane. Filtrate was inoculated into Vero cells and the cells were incubated at 37 °C for 18~24 h to observe the morphology. Filtrates collected from STEC O157:H7 strain EDL933, *E. coli* DH5α, cell medium were included as controls.

### Antimicrobial susceptibility tests

Antimicrobial susceptibility of the STEC isolates was determined by using the disc diffusion method, following the protocols recommended by Clinical and Laboratory Standards Institute [74]. A total of 14 types of antibiotics including amoxicillin (AMX), ampicillin (AMP), sulfafurazole (SIX), streptomycin (STM), norfloxacin (NRF), gentamicin (GEN), tetracycline (TET), neomycin (NEO), doxycycline (DOX), trimethoprim-sulfamethoxazole (SXT), kanamycin (KAN), cefotaxime (CFX), erythromycin (ERY), and ofloxacin (OFX) were tested. Results were interpreted using the CLSI breakpoints, when available. Resistance to colistin was also tested using broth microdilution method, as recommended by CLSI [74]. Colistin with final concentrations of 1 μg/mL, 2 μg/mL, and 4 μg/mL was made in a 96-well plate in pre-reduced supplemented Mueller-Hinton (MH) broth (Hopebio, Qingdao, China). Interpretation of testing results was based on EUCAST breakpoint (> 2 μg/mL), as the CLSI document (VET01S) does not provide a breakpoint for interpretation of colistin. Each antibiotic was tested with three duplicates. *E. coli* ATCC^R^ 25922 was used as quality control.

### Multilocus sequence typing

Multilocus sequence typing (MLST) was performed using the previously described protocols [60]. Nucleotide sequences of seven housekeeping genes (*adk*, *fumC*, *gyrB*, *icd*, *mdh*, *purA*, and *recA*) were amplified and sequenced using the primers listed in Table 2. PCR reaction was performed in a 50-μl reaction mixture containing 2 μl of the template DNA, 2 μL of dNTP mixture (TAKARA, Japan), 5 μL of 10 × PCR buffer (TAKARA, Japan), 0.5 μL of rTaq polymerase (TAKARA, Japan), each of the forward and reverse primer 1 μL, and 38.5 μL of nuclease-free water. The reaction was performed under the following standard cycling procedure: an initial denaturation at 95 °C for 5 min, followed by 30 cycles of denaturation at 94 °C for 30 s, annealing at 54–60 °C for 45 s (see Table 2), extension at 72 °C for 1 min, and a final extension at 72 °C for 10 min. The PCR products were initially analyzed by electrophoresis on a 1% agarose gel. Products with the correct size were sequenced at Sangon (Shanghai, China). Nucleotide sequences of the housekeeping genes were submitted to the *Escherichia coli* MLST Database (http://mlst.warwick.ac.uk/mlst/dbs/Ecoli) to determine the sequence types automatically. Phylogenetic tree was generated based on the MLST data by MEGAX [26], using neighbor-joining algorithm with 1000 bootstrapping.

## Data Availability

Not applicable.

## Abbreviations

AMP: Ampicillin
CFX: Cefotaxime
DOX: Doxycycline
ERY: Erythromycin
GEN: Gentamicin
KAN: Kanamycin
LEE: The locus of enterocyte effacement
LPS: Lipopolysaccharide
MLST: Multilocus sequence typing
NEO: Neomycin
NRF: Norfloxacin
OFX: Ofloxacin
OMPs: Outer membrane proteins
Saa: STEC autoagglutinating adhesion
SIX: Sulfafurazole
ST: Sequence type
STEC: Shiga toxin-producing *Escherichia coli*
STM: Streptomycin
Stx: Shiga toxin
*stx*: Shiga toxin encoding genes
SXT: Trimethoprim-sulfamethoxazole
TET: Tetracycline
